# Gut microbiota and bile acid profiles in purebred vs. crossbred sows: links to oxidative stress and inflammation in late gestation

**DOI:** 10.1099/mgen.0.001579

**Published:** 2025-12-03

**Authors:** Chenggang Yin, Lei Xu, Zixi Wei, Ying Zhao, Rong Bai, Ge Gao, Yuyang Fan, Yanpin Li, Wenjuan Sun, Xilong Li, Yu Pi

**Affiliations:** 1 Key Laboratory of Feed Biotechnology of Ministry of Agriculture and Rural Affairs, Institute of Feed Research, Chinese Academy of Agriculture Sciences, Beijing 100081, PR China; 2 Precision Livestock and Nutrition Unit, TERRA Teaching and Research Centre, Gembloux Agro-Bio Tech, University of Liège, Gembloux 5030, Belgium; 3 Department of Business Economics, Wageningen University, 6700 EW Wageningen, The Netherlands

**Keywords:** bile acid metabolism, crossbreeding, gut microbiota, late gestation, oxidative stress, sows

## Abstract

Understanding the interactions between gut microbiota, bile acid (BA) metabolism and systemic health is critical for supporting gestational physiological stability in sows, especially during the physiologically demanding late gestation period. Although physiological advantages vary by breed in late-gestation sows, the microbiota-related mechanisms underlying these differences remain poorly understood. This study compared serum antioxidant enzyme activity, oxidative damage markers, inflammatory cytokine levels, gut microbiota composition (analysed via 16S rRNA sequencing), and BA profiles (assessed through targeted metabolomics) between purebred large white (LW) and large white×landrace (LW×LR) crossbred sows during late gestation. Results showed that LW×LR crossbred sows exhibited significantly higher serum superoxide dismutase (SOD) activity and IL-10 levels, alongside reduced IL-6 levels (*P*<0.05), indicating enhanced antioxidant and anti-inflammatory capacity. Gut microbiota analysis revealed greater alpha diversity (Shannon indices) and a lower Simpson index, along with distinct beta diversity (*P*<0.05) in crossbred sows, with notable enrichment of functional taxa such as *Treponema* and *Prevotella*. Additionally, faecal concentrations of modified BAs, specifically 3-oxolithocholic acid and 7-ketolithocholic acid, were significantly elevated, correlating with increased abundance of gut microbiota encoding BA: Na^+^ symporter (BASS family) proteins, as well as the increased 7-*α*-hydroxysteroid dehydrogenase activity (*P*<0.05). In contrast, LW sows exhibited enrichment of *Terrisporobacter* and *Clostridium sensu* stricto 1, alongside accumulation of primary (e.g. chenodeoxycholic acid) and unconjugated BAs (e.g. deoxycholic acid) (*P*<0.05). Correlation analysis demonstrated that the accumulation of *Terrisporobacter* and primary BAs was positively correlated with exacerbation of inflammation. In conclusion, under intensive production conditions, significant differences in the gut microbiota–BA axis between LW and LW×LR crossbred sows may underlie variations in oxidative stress and inflammatory status during late pregnancy. These findings provide valuable insights into microbiome–BA–host associations underlying the physiological advantages (enhanced antioxidant and anti-inflammatory capacity) of crossbred sows.

## Data Summary

The microbiome sequencing data have been deposited in the National Center for Biotechnology Information (NCBI) database, accessible through BioProject accession number PRJNA1295093, which contains the NCBI Biosamples (Table S1). Data on faecal bile acid profiles and blood biochemical indicators are publicly available at the Open Science Framework repository (https://osf.io/kqtav).

**Impact Statement**

This study highlights the regulatory role of genetic background on gestational adaptation in sows through the gut microbiota–bile acid (BA)–host interaction axis. Compared to purebred large white (LW) sows, large white×landrace crossbred sows exhibited enhanced antioxidant and anti-inflammatory capacities in late pregnancy, accompanied by enrichment of beneficial bacteria such as *Prevotella*, elevated levels of regulatory BAs (e.g. 3-oxolithocholic acid) and increased expression of BA transporters. These features collectively contributed to improved metabolic and immune stability. In contrast, purebred LW sows showed signs of proinflammatory microbiota enrichment and BA accumulation, potentially disrupting physiological homeostasis. These findings provide insights into gut microbiota–BA–host crosstalk underlying the physiological advantages of crossbreeding and underscore the value of genetic diversity in enhancing gestational metabolic and immune stability. The results also offer a scientific basis for targeted strategies aimed at improving sow health and productivity, such as optimizing crossbreeding programmes and developing interventions that modulate the gut microbiota and BA metabolism.

## Introduction

As the cornerstone of swine production, the physiological stability of sows plays a pivotal role in determining farming efficiency. Due to genetic variation, sows of different breeds or crossbreeding combinations often exhibit significant variation in growth performance, gestational physiological adaptability and overall health status [1,4]. Large white (LW) sows, a widely raised purebred breed, are globally renowned for their rapid growth rate, high feed conversion efficiency and elevated lean meat percentage [5], making them central to intensive pig production systems. However, as production demands continue to rise, the limitations of single purebred lines have become more apparent. In this context, two-way crossbred sows derived from large white×landrace (LW×LR) breeds have gained prominence as maternal lines in large-scale operations, exhibiting heterosis in stress resistance, adaptability and overall production performance compared to their purebred counterparts [6,7]. Despite these practical advantages, the physiological mechanisms underlying these genotype-based differences remain poorly understood.

Late gestation constitutes a critical period for physiological regulation in sows, characterized by intensified fetal growth and elevated maternal nutrient demands [8], which impose a substantial metabolic burden [9], thereby triggering oxidative stress and immunological challenges [10,11]. During this period, maintaining maternal homeostasis relies heavily on the dynamic balance between antioxidant defences and immune regulation, both of which are closely associated with reproductive outcomes and maternal health. A balanced gut microbiota plays a critical role in maintaining host metabolic homeostasis and regulating immune system development. Numerous studies have demonstrated that through influencing nutrient absorption, energy balance and immune homeostasis, gut microbial communities are integral to maternal well-being during pregnancy [12,13]. Shifts in microbial composition, particularly during late gestation, have been linked to changes in host physiological status (e.g. oxidative stress and inflammation) [14] and are often characterized by overrepresentation of *Proteobacteria* and *Actinobacteria* [15], which are associated with inflammation and metabolic disturbances [13]. Gut microbiota dysbiosis can lead to impaired digestion and disrupted nutrient utilization [16], thereby reducing the efficiency of nutrient intake and assimilation and ultimately limiting the availability of essential nutrients to the fetus. Moreover, maternal microbiota imbalances have transgenerational consequences, affecting not only the maternal immune milieu but also offspring microbial colonization and immune development [17]. In addition, the metabolic activity of the gut microbiota produces a wide range of bioactive compounds, among which BAs serve as key metabolites that exert pleiotropic regulatory effects on various host physiological processes.

Among the numerous microbial metabolites influencing host physiology, BAs represent a key class of bioactive molecules that mediate host–microbiota interactions. Synthesized in the liver and secreted into the intestine, BAs facilitate lipid metabolism and the absorption of fat-soluble vitamins [18,19], while also participating in systemic metabolic and immune regulation by activating nuclear receptors [e.g. farnesoid X receptor (FXR)] and membrane-bound receptors (e.g. Takeda G protein-coupled receptor 5/membrane-bound BAR domain protein) [20,22]. The gut microbiota can convert primary BAs into secondary and modified forms via deconjugation and dehydrogenation reactions, establishing a bidirectional interaction that shapes intestinal health, metabolic homeostasis and immune equilibrium [23,25]. Emerging evidence highlights the pivotal roles of specific BAs in shaping gut microbial ecology and modulating host metabolism and immunity. For instance, ursodeoxycholic acid (UDCA) has been shown to enhance embryo implantation and metabolic health by modulating maternal gut microbiota–metabolite interactions [26], while also improving intestinal barrier function in low birth weight newborn piglets [27]. Additionally, gut microbiota–derived metabolites such as 12-ketolithocholic acid (12-ketoLCA) and hyodeoxycholic acid (HDCA) have demonstrated significant anti-inflammatory effects, particularly in the models of ulcerative colitis [28,29]. Conversely, certain BAs, such as lithocholic acid (LCA) and deoxycholic acid (DCA), negatively impact porcine intestinal epithelial cell proliferation and barrier function [30,31]. Excessive BA accumulation in the gut may further compromise intestinal barrier integrity and trigger mucosal inflammation [32].

Although previous studies have investigated the gut microbiota, BA metabolism and markers of oxidative stress and inflammation in pigs, most have focused on single parameters or specific physiological stages. Comprehensive comparisons between LW sows and LW×LR crossbred sows during late gestation remain limited. To address this gap, the present study systematically compared serum antioxidant and inflammatory indicators, faecal gut microbiota composition and BA metabolic profiles between LW and LW×LR crossbred sows in late pregnancy. Furthermore, potential interactions among these factors were explored to identify key physiological differences and regulatory mechanisms, thereby providing a theoretical basis for understanding functional divergence between pig breeds.

## Methods

### Animal ethics approval

The animal protocol in this study was approved by the Animal Care and Use Committee of the Institute of Feed Research of the Chinese Academy of Agricultural Sciences (IFR-CAAS20240618).

### Experimental animals and treatment

Twenty healthy LW sows and twenty-four healthy LW×LR crossbred sows were randomly selected from the same large-scale commercial pig farm. All sows met strict inclusion criteria: good body condition, body weight ranging from 220 to 250 kg, similar parity (third to fourth parity), no history of reproductive diseases in the past 6 months and no antibiotic use within 4 weeks before the experiment. The experiment was conducted as a single independent batch. This design was determined based on the large number of sows in each group and standardized on-farm management conditions (which minimized batch-to-batch variation). They were housed in individual gestation stalls, provided ad libitum access to water and fed the same gestation-phase complete diet formulated to meet the nutrient requirements of swine as recommended by the National Research Council (NRC) (2012). The feed formula and nutrient composition table are shown in Table S2 (available in the online Supplementary Material). The sows were fed twice daily at 08:00 AM and 04:00 PM. The feed allowance was 2.5 kg per sow per day. The housing environment was maintained with good ventilation, with temperatures controlled at 20–25 °C and relative humidity maintained between 60 and 70%.

### Sample collection

On day 90 of gestation, 10 ml of fasting blood was collected using sterile, non-anticoagulant vacuum tubes. After standing at room temperature for 30 min, samples were centrifuged at 3,000 r.p.m. for 15 min to isolate serum, which was aliquoted into 1.5- ml tubes and stored at −20 °C for antioxidant and inflammatory factor analysis. Approximately 50 g of fresh faecal sample was collected from each sow on the same day, before morning feeding. This timing was chosen to reduce the potential influence of food intake on faecal BA variability. Samples were immediately transferred to sterile centrifuge tubes and stored at −80 °C for 16S rRNA gene sequencing and targeted BA metabolomics.

### Serum antioxidant enzyme activity and inflammatory cytokine measurements

Serum total antioxidant capacity (T-AOC), superoxide dismutase (SOD), catalase (CAT) activity and malondialdehyde (MDA) content were measured using commercial assay kits. The levels of IL-6, IL-1*β*, tumour necrosis factor-*α* (TNF-*α*) and IL-10 in serum were determined using ELISA kits. The antioxidant assay kits were purchased from Nanjing Jiancheng Bioengineering Institute (Nanjing, China), and the ELISA kits were obtained from Shanghai Enzyme-linked Biotechnology Co., Ltd. (Shanghai, China). All procedures were performed following the manufacturers’ instructions and previously published methods [33].

### 16S rRNA gene sequencing analysis of faecal microbiome

Following the procedures described in previous studies [34], total microbial DNA was isolated from frozen faecal samples using the Faecal Genomic DNA Extraction Kit (Omega Bio-Tek, Norcross, GA, USA). DNA integrity was assessed by 1% agarose gel electrophoresis, and its concentration and purity were measured using a NanoDrop™ One spectrophotometer (Thermo Fisher Scientific, Waltham, MA, USA) via absorbance at 260 nm and 280 nm (A260/A280 ratio). DNA with an A260/A280 ratio of 1.8–2.0 (high purity, free from contaminants), intact bands in 1% agarose gel (no degradation) and concentration ≥50 ng µl^−1^ (sufficient for PCR) was used as a template for PCR amplification targeting the V3–V4 hypervariable region of the bacterial 16S rRNA gene using specific primers 338F (5′-ACTCCTACGGGAGGCAGCAG-3′) and 806R (5′-GGACTACHVGGGTWTCTAAT-3′) [33]. The PCR products were purified (Axygen Biosciences, Union City, CA, USA), quantified and used for library construction. Paired-end sequencing (2×250 bp) was conducted on the Illumina MiSeq platform. Raw sequencing data were denoised using DADA2 (version 1.26.0) in QIIME 2 (v2022.2) with default parameters to generate amplicon sequence variants (ASVs), which were taxonomically annotated using the silva 138.2 reference database [35]. Downstream analyses, sequences were first rarefied to the minimum number of sequences per sample. Subsequently, analyses including taxonomic classification, community diversity (*α-*/*β*-diversity), species difference (e.g. ANOSIM), correlation (Pearson), phylogenetic analysis and functional prediction (PICRUSt2) were performed using the Majorbio Cloud Platform (https://cloud.majorbio.com/). Beta diversity was assessed by principal coordinate analysis (PCoA) based on Bray–Curtis, weighted UniFrac and unweighted UniFrac distances, the latter two calculated from a FastTree (v2.1.11) phylogeny. Community differences were tested with analysis of similarities (ANOSIM). Microbial community assembly processes were evaluated using the neutral community model (NCM) fitted with ASV relative abundances; goodness of fit was estimated by *R*², with ASV distribution classified according to 95% bootstrap confidence intervals. Functional prediction was conducted with PICRUSt2 based on 16S rRNA gene data, and Kyoto Encyclopedia of Genes and Genomes (KEGG) pathway annotations were used to infer functional profiles. The raw sequencing data have been deposited in the National Center for Biotechnology Information Sequence Read Archive under the accession number PRJNA1295093.

### Faecal BA profile detection

Targeted metabolomic profiling of BAs was performed by a commercial service provider (Metabolo-Profile Biotechnology, Shanghai, China). The experimental procedure was conducted according to a previously reported method [36]. Briefly, after sample pretreatment, analysis was carried out using a Waters ACQUITY UPLC system coupled with an Xevo G2-S Q-TOF high-resolution mass spectrometer (Waters Corp., Milford, MA, USA). Ionization was achieved via electrospray ionization (ESI) operated in negative ion mode. Chromatographic separation was performed on an ACQUITY BEH C18 column (1.7 µm, 100 mm × 2.1 mm; Waters Corp., Milford, MA, USA), with an injection volume of 5 µl, a column temperature of 35 °C and a flow rate of 0.45 ml min^−1^ under a gradient elution programme. Mass spectrometric data were acquired in multiple reaction monitoring mode, with a scan time of 0.036 s/scan. Raw data were collected and processed using MassLynx software (v4.1, Waters Corp., Milford, MA, USA). Identification and quantification of BAs were performed using external standards.

### Statistical analysis

Alpha diversity (Shannon, Simpson) and the relative abundances of dominant taxa (phylum and genus) were compared between groups using the Wilcoxon rank-sum test. Serum antioxidant parameters, inflammatory cytokines and faecal BA concentrations were analysed in SPSS (v24.0). Normality and homogeneity of variance were tested before applying independent-sample t-tests. Pearson correlation analysis was performed among serum oxidative stress markers, cytokines, gut microbiota (genus level) and faecal BAs, with correlation heatmaps generated in R (v4.4.2). Statistical significance was defined as *P<0.05, **P<0.01 and ***P<0.001.

## Results

### Variations of serum oxidative stress and inflammation-related cytokine indicators in LW and LW×LR crossbred sows at late pregnancy

**Fig. 1.**
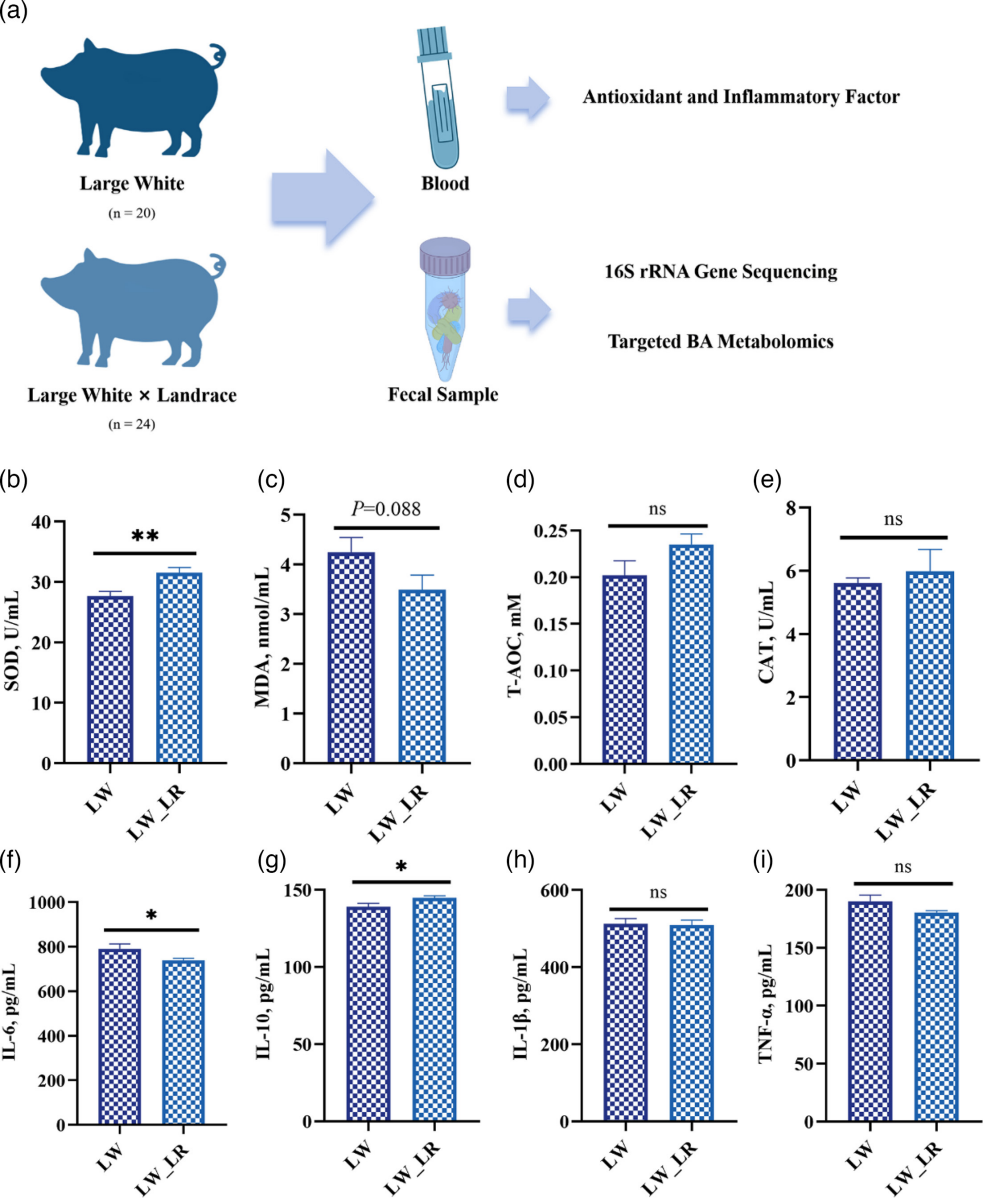
Experimental schematic diagram (**a**) and changes in serum oxidative stress and inflammation-related markers in LW and LW×LR crossbred sows during late pregnancy (**b–i**). LW_LR, large white×landrace crossbred sows; ns, No significance; *P<0.05; **P<0.01.

To compare serum oxidative stress indices and inflammation-related cytokine indicators levels between LW sows and LW×LR crossbred sows during late gestation, relevant parameters were measured using commercial assay kits. In the late gestation, compared with LW sows, LW×LR sows exhibited significantly higher serum SOD activity (*P*<0.05, Fig. 1b) and elevated levels of the anti-inflammatory cytokine IL-10 (*P*<0.05, Fig. 1g), along with a significant reduction in the pro-inflammatory cytokine IL-6 (*P*<0.05, Fig. 1f). Additionally, serum MDA concentrations in LW×LR sows were lower than those in LW sows (*P*=0.088, Fig. 1c). No significant differences were observed between groups in terms of T-AOC, CAT activity, IL-1*β* or TNF-*α* levels (*P*>0.05, Fig. 1d, e, h and i).

### Comparison of faecal microbial community composition between LW and LW×LR crossbred sows at late pregnancy

**Fig. 2.**
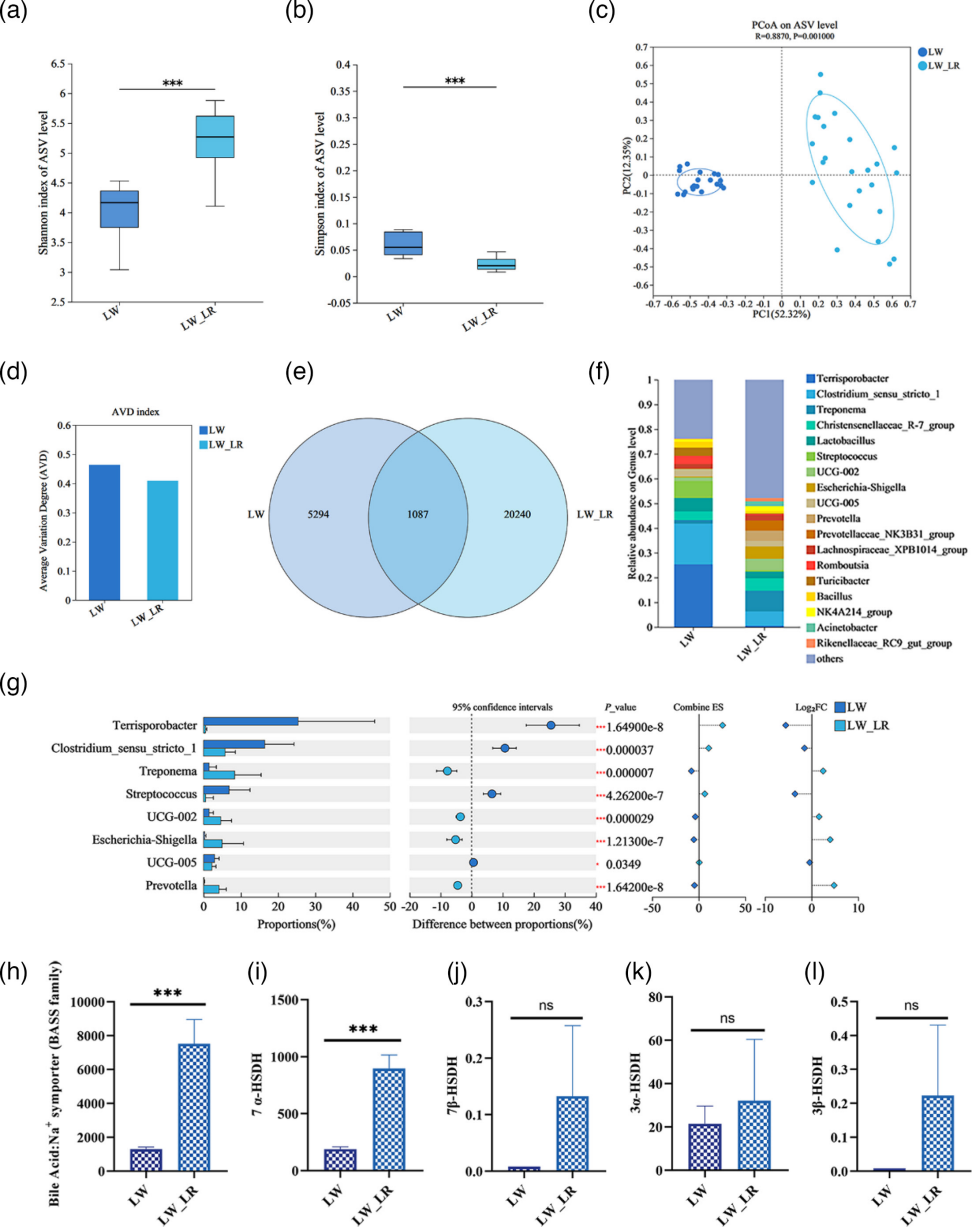
Faecal microbiota analysis in LW and LW×LR crossbred sows during late pregnancy. (**a**) Shannon index; (**b**) Simpson index; (**c**) PCoA was performed based on weighted UniFrac distances at the ASVs level to assess *β*-diversity; (**d**) Microbial community stability index was assessed using the AVD, which serves as a measure of bacterial community stability. (**e**) Venn diagram of shared ASVs; (**f**) Microbial composition at the genus level; (**g**) Differential analysis of genus-level microbial composition; (**h**) KEGG-based functional prediction-BA: Na^+^ symporter, BASS family, (**i**) 7*α*-hydroxysteroid dehydrogenase, (**j**) 7*β*-hydroxysteroid dehydrogenase, (**k**) 3*α*-hydroxysteroid dehydrogenase and (**l**) 3*β*-hydroxysteroid dehydrogenase. LW_LR, large white×landrace crossbred sows; ns, no significance; ***P<0.001.

To compare the gut bacterial community composition between LW and LW×LR crossbred sows, 16S rRNA gene sequencing was performed. The rarefaction curves (Fig. S1) indicate that sequencing depth was sufficient for all samples, as the curves plateaued, suggesting adequate coverage of the microbial diversity. Regarding alpha diversity, the Shannon indices of faecal microbiota in LW×LR crossbred sows were significantly higher than those in LW sows, while the Simpson index was significantly lower in LW×LR crossbred sows (*P*<0.05, Fig. 2a,b). PCoA based on weighted UniFrac, unweighted UniFrac and Bray–Curtis distances revealed clear segregation of faecal microbiota at the ASV level between LW×LR crossbred sows and LW sows (weighted UniFrac: ANOSIM *P*<0.05, *R*=0.887, Fig. 2c; unweighted UniFrac: ANOSIM *P*<0.05, *R*=0.968, Fig. S2A; Bray–Curtis: ANOSIM *P*<0.05, *R*=0.941, Fig. S2B). Analysis of the microbial stability index [average variation degree (AVD)] revealed that LW×LR crossbred sows exhibited a lower AVD value compared to LW sows (0.41 vs. 0.46), indicating greater gut microbiota stability during late gestation (Fig. 2d). NCM analysis showed that the faecal microbiota of LW and crossbred LW×LR sows exhibited *R*² values of 0.68 and 0.64, respectively, with corresponding migration rates (M) of 0.0155 and 0.0163. These results suggest potential differences in community assembly processes between the two groups (Fig. S3).

These results indicate that LW×LR crossbred sows possess a gut microbiota with greater richness and diversity. Venn diagram analysis showed that 1,087 ASVs were shared between LW and LW×LR crossbred sows. The number of unique ASVs was 2,345 in LW×LR crossbred sows and 1,568 in LW sows (Fig. 2e). Analysis of community composition at the phylum level revealed that both LW and LW×LR crossbred sows faecal microbiota were dominated by *Firmicutes* (90.2% and 46.1%, respectively), *Bacteroidota* (4.9% and 34.9%), *Spirochaetota* (1.5% and 8.7%) and *Proteobacteria* (1.3% and 7.1%) (Fig. S4A). The *Firmicutes*/*Bacteroidota* ratios in LW sows and LW×LR crossbred sows were 18.61 and 1.32, respectively. Similarly, phylum-level differential analysis (Fig. S4B) revealed that crossbred LW×LR sows exhibited lower relative abundances of *Firmicutes* and *Actinobacteriota*, but higher abundances of *Bacteroidota*, *Spirochaetota* and *Proteobacteria* compared to LW sows (*P*<0.05).

At the genus level, the three most relatively abundant genera in both groups were *Terrisporobacter*, *Clostridium sensu* stricto 1 and *Treponema* (Fig. 2f). Specifically, the relative abundances of *Terrisporobacter* were 25.4% in LW sows and 0.6% in LW×LR crossbred sows; *Clostridium sensu* stricto 1 accounted for 16.4% and 5.8%, respectively; *Treponema* accounted for 1.5% and 8.4%, respectively. Genus-level differential analysis revealed that the relative abundances of *Treponema*, *UCG-002* (*Oscillospiraceae* family), *Escherichia–Shigella* and *Prevotella* were significantly higher in the faecal microbiota of LW×LR crossbred sows compared to LW sows. Conversely, *Terrisporobacter*, *Clostridium sensu* stricto 1, *Streptococcus* and *UCG-005* (*Oscillospiraceae* family) were significantly more abundant in LW sows (*P*<0.05, Fig. 2g).

Functional prediction analysis based on KEGG pathway abundances (Fig. S5A) and enzyme abundance (Fig. S5B) showed that the predicted abundance of the ‘BA: Na^+^ symporter (BASS family)’ gene pathway was significantly higher in LW×LR crossbred sows compared to LW sows (*P*<0.05, Fig. 2h). In comparison, the expression of 7-*α*-hydroxysteroid dehydrogenase (7*α*-HSDH), an enzyme involved in BA metabolism, was significantly increased in LW×LR crossbred sows (*P*<0.05, Fig. 2i). Additionally, there were no significant differences in the expressions of 7-*β*-hydroxysteroid dehydrogenase, 3-*α*-hydroxysteroid dehydrogenase (3*α*-HSDH), and 3-*β*-hydroxysteroid dehydrogenase (*P*>0.05, Fig. 2j–l).

### Comparison of faecal BA profiles between LW and LW×LR crossbred sows

**Fig. 3.**
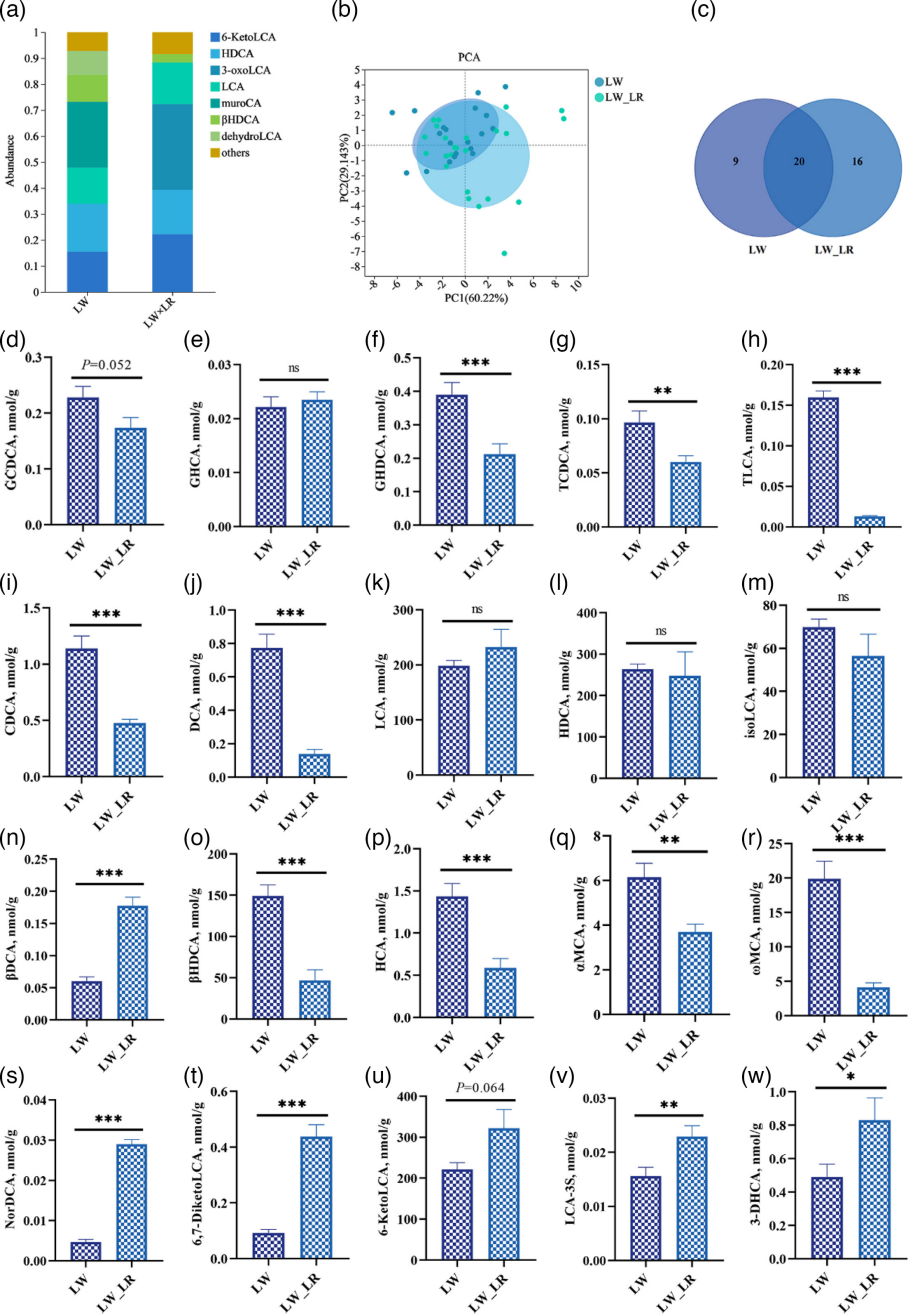
Faecal BA composition and shared BA analysis in LW and LW×LR crossbred sows during late pregnancy. (**a**) Composition of BAs; (**b**) PCA; (**c**) Venn diagram showing shared BAs species; (d–w) Composition of shared faecal BAs in LW and LW×LR crossbred sows during late pregnancy. GCDCA, glycochenodeoxycholic acid; GHCA, glycohyocholic acid; GHDCA, glycohyodeoxycholic acid; TCDCA, taurochenodeoxycholic acid; TLCA, taurolithocholic acid; CDCA, chenodeoxycholic acid; isoLCA, isolithocholic acid; *β*DCA, *β*-deoxycholic acid; βHDCA, *β*-hyodeoxycholic acid; HCA, hyocholic acid; αMCA, *α*-muricholic acid; *ω*MCA, *ω*-muricholic acid; NorDCA, 23-Nordeoxycholic acid; 6,7-DiketoLCA, 6,7-diketolithocholic acid; LCA-3S, lithocholic acid 3-sulphate; 3-DHCA, 3-dehydrocholic acid; LW_LR, large white×landrace crossbred sows; ns, no significance; *P<0.05; **P<0.01; ***P<0.001.

**Fig. 4.**
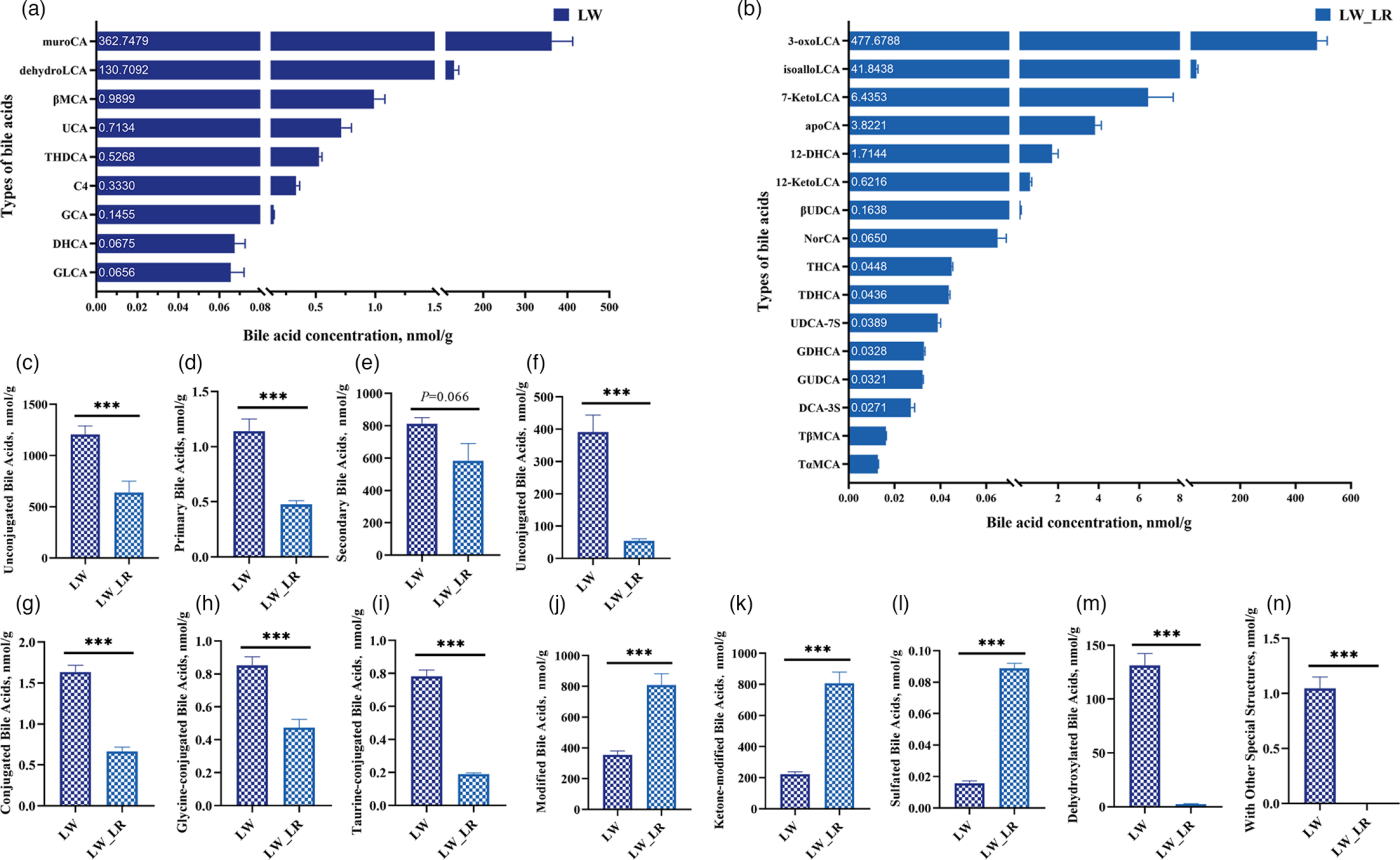
Differential and unique faecal BAs and their classifications in LW and LW×LR crossbred sows during late pregnancy. (**a**) Unique BAs in LW sows: muroCA, dehydroLCA, *β*MCA, UCA, THDCA, C4, GCA, DHCA and GLCA; (**b**) Unique BAs in LW×LR crossbred sows: 3-oxoLCA, isoalloLCA, I7-KetoLCA, apoCA, 12-KetoLCA, βUDCA, NorCA, THCA, TDHCA, UDCA-7S, GDHCA, GUDCA, DCA-3S, T*β*MCA and T*α*MCA; (**c**) Unconjugated BAs; (**d**) Primary BAs; (**e**) Secondary BAs; (**f**) Other unconjugated BAs include HCA, muroCA, αMCA, βMCA, ωMCA, apoCA, NorCA, NorDCA and isoalloLCA; (**g**) Conjugated BAs; (**h**) Glycine-conjugated BAs; (**i**) Taurine-conjugated BAs; (**j**) Modified BAs; (**k**) Keto-modified BAs; (**l**) Sulphated BAs; (**m**) Dehydrogenated BAs; (**n**) Other structurally special BAs include UCA and C4. LW_LR, large white×landrace crossbred sows; ***P<0.001.

To compare faecal BA composition between LW and LW×LR crossbred sows, targeted analysis of BA species and concentrations was performed using HPLC coupled with ESI-MS/MS. The results showed that 3-oxolithocholic acid (3-oxoLCA), 6-ketolithocholic acid (6-KetoLCA) and HDCA exhibited the highest concentrations in the faeces of LW×LR crossbred sows, whereas murocholic acid (muroCA), HDCA and 6-KetoLCA were most abundant in LW sows (Fig. 3a). Principal component analysis (PCA) revealed no clear separation in faecal BA profiles between the two groups (Fig. 3b). A total of 45 BA species were detected in the faecal samples, of which 20 were shared between LW and LW×LR crossbred sows (Fig. 3c). LW sows exhibited nine unique BAs, including muroCA, dehydrolithocholic acid (dehydroLCA), *β*-muricholic acid (*β*MCA), ursocholic acid (UCA), taurohyodeoxycholic acid (THDCA), 7*α*-hydroxy-cholestene-3-one (C4), glycocholic acid (GCA), dehydrocholic acid (DHCA) and glycolithocholic acid (GLCA) (Fig. 4a). However, LW×LR crossbred sows had 16 unique BAs, such as 3-oxoLCA, isoallolithocholic acid (isoalloLCA), 7-ketolithocholic acid (7-KetoLCA), apocholic acid (apoCA), 12-ketolithocholic acid (12-KetoLCA), 3*β*-ursodeoxycholic acid (*β*UDCA), norcholic acid (NorCA), taurohyocholic acid (THCA), taurodehydrocholic acid (TDHCA), ursodeoxycholic acid-7 sulphate (UDCA-7S), glycodehydrocholic acid (GDHCA), glycoursodeoxycholic acid (GUDCA), doxycholic acid-3 sulphate (DCA-3S), tauro-*β*-muricholic acid (T*β*MCA) and tauro-*α*-muricholic acid (T*α*MCA) (Fig. 4b).

Differential BA analysis revealed that the concentrations of glycohyodeoxycholic acid (GHDCA) (Fig. 3f), taurochenodeoxycholic acid (Fig. 3g), taurolithocholic acid (TLCA) (Fig. 3h), chenodeoxycholic acid (CDCA) (Fig. 3i), DCA (Fig. 3j), *β*-hyodeoxycholic acid (βHDCA) (Fig. 3o), hyocholic acid (HCA) (Fig. 3p), *α*-muricholic acid (*α*MCA) (Fig. 3q) and *ω*-muricholic acid (*ω*MCA) (Fig. 3r) were significantly higher in the faeces of LW sows compared to LW×LR crossbred sows. Conversely, levels of *β*-deoxycholic acid (*β*DCA) (Fig. 3n), 23-nordeoxycholic acid (NorDCA) (Fig. 3s), 6,7-diketolithocholic acid (6,7-DiketoLCA) (Fig. 3t), lithocholic acid 3-sulphate (LCA-3S) (Fig. 3v) and 3-dehydrocholic acid (Fig. 3w) were significantly lower in LW sows than in LW×LR crossbred sows (*P*<0.05).

Further classification analysis of BAs revealed that the concentrations of both unconjugated (Fig. 4c) and conjugated BAs (Fig. 4g) were significantly higher in the faeces of LW sows compared to LW×LR crossbred sows. This difference was mainly attributed to elevated levels of primary BAs (Fig. 4d), other unconjugated BAs (Fig. 4f), glycine-conjugated BAs (Fig. 4h) and taurine-conjugated BAs (Fig. 4i) in LW sows (*P*<0.05). Conversely, modified BAs (Fig. 4j) were significantly more abundant in LW×LR crossbred sows, with higher levels of keto-modified BAs (Fig. 4k), sulphated BAs (Fig. 4l), dehydrogenated BAs (Fig. 4m) and other structurally distinct BAs (Fig. 4n) compared to LW sows (*P*<0.05).

### Correlations between oxidative stress, inflammation, gut microbiota and faecal BAs

**Fig. 5.**
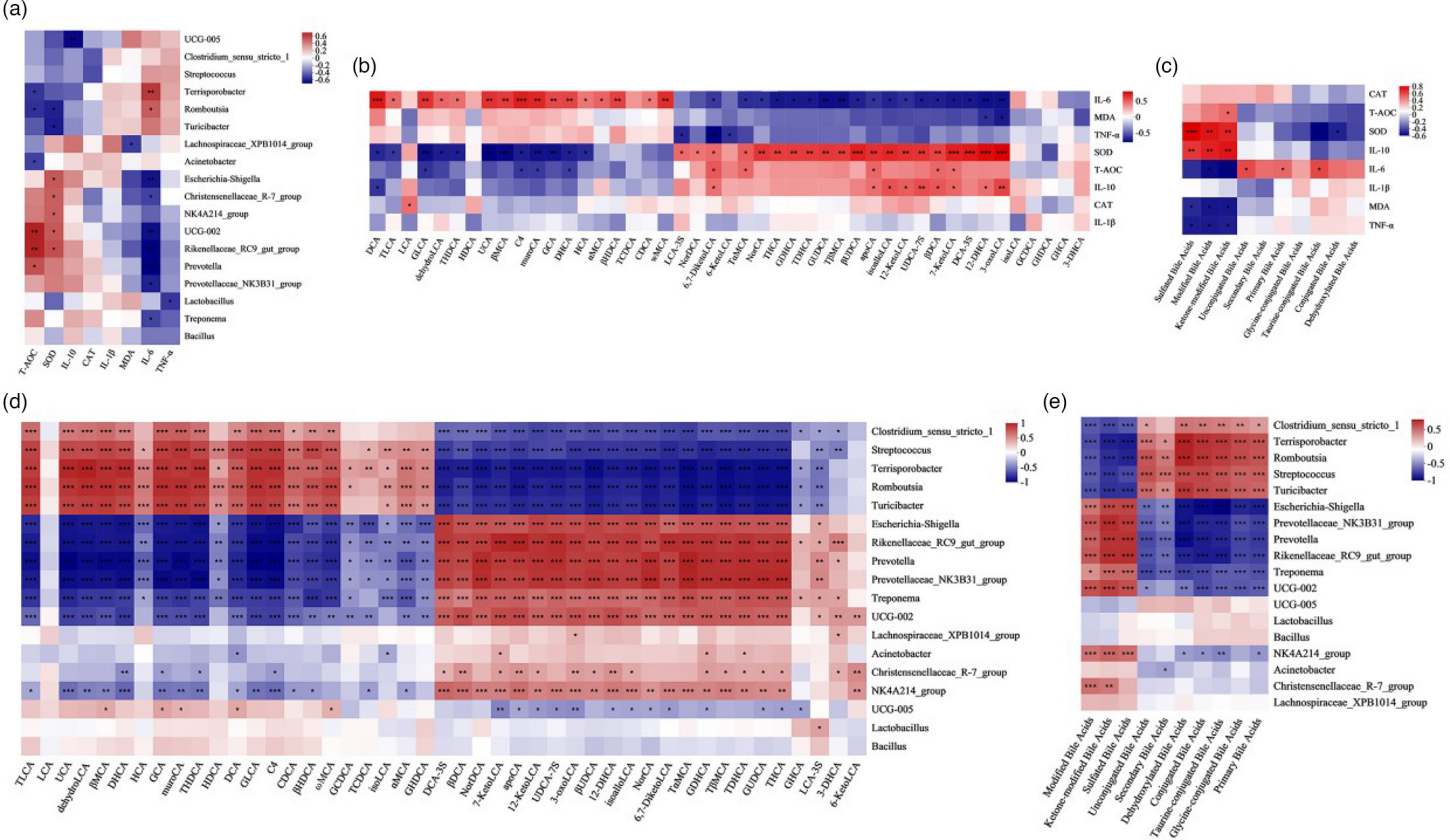
Correlation analysis between serum oxidative stress and inflammatory markers, faecal microbiota at the genus level and BAs profiles in LW and LW×LR crossbred sows during late pregnancy. (**a**) Correlations between serum oxidative stress and inflammatory markers and faecal microbiota at the genus level; (**b, c**) Correlations between serum oxidative stress and inflammatory markers and faecal BAs composition; (**c, d**) Correlations between faecal microbiota at the genus level and faecal BAs composition. LW_LR, large white×landrace crossbred sows; *P<0.05; **P<0.01; ***P<0.001.

To further explore the potential relationships between serum oxidative stress, inflammatory markers and faecal microbiota in late-gestation sows, correlation analysis was performed. SOD activity was positively correlated with the abundances of *Escherichia–Shigella* and *UCG-002* (*Oscillospiraceae* family) (*P*<0.05, Fig. 5a). IL-6 was positively correlated with *Terrisporobacter* and negatively correlated with *Prevotella*, *UCG-002* (*Oscillospiraceae* family), and *Treponema* (*P*<0.05, Fig. 5a). IL-10 showed a negative correlation with *UCG-005* (*Oscillospiraceae* family) abundance (*P*<0.05, Fig. 5a).

Correlation analysis between serum markers and faecal BAs revealed that SOD activity positively correlated with LCA-3S, NorDCA, 6,7-DiketoLCA, *β*DCA, 3-oxoLCA and various modified BAs, but negatively correlated with TLCA, dehydroLCA, muroCA, conjugated BAs and taurine-conjugated BAs (*P*<0.05, Fig. 5b, c). MDA levels negatively correlated with 3-oxoLCA and several modified BAs (*P*<0.05, Fig. 5b, c). IL-6 levels were negatively associated with 6,7-DiketoLCA, *β*DCA, 3-oxoLCA and keto- and sulphated BAs, but positively correlated with DCA, TLCA, dehydroLCA, muroCA, HCA, *α*MCA, *β*HDCA, CDCA, ωMCA, unconjugated, primary and taurine-conjugated BAs (*P*<0.05, Fig. 5b, c). IL-10 was negatively correlated with DCA and positively with several modified BAs (*P*<0.05, Fig. 5b, c).

Further, the faecal microbiota genera *Treponema*, UCG-002 (*Oscillospiraceae* family), *Escherichia–Shigella* and *Prevotella* exhibited positive correlations with the levels of specific BAs, including 3-oxoLCA, 6,7-DiketoLCA, isoalloLCA, LCA-3S, NorDCA and *β*DCA, as well as modified BAs, ketone-modified BAs and sulphated BAs. In contrast, they were negatively correlated with the levels of GHDCA, TLCA, CDCA, DCA, dehydroLCA, muroCA, *ω*MCA, *α*MCA, *β*HDCA and the BA subgroups of conjugated BAs (including glycine-conjugated and taurine-conjugated BAs), unconjugated BAs, primary BAs and dehydroxylated BAs (*P*<0.05, Fig. 5d, e). Conversely, *Terrisporobacter* and *Clostridium sensu stricto* 1 showed the opposite correlation patterns: they were positively correlated with conjugated BAs (glycine-conjugated and taurine-conjugated BAs), unconjugated BAs, primary BAs and dehydroxylated BAs, but negatively correlated with modified BAs, ketone-modified BAs and sulphated BAs (*P*<0.05, Fig. 5d, e). Additionally, the abundance of UCG-005 (*Oscillospiraceae* family) was positively correlated with individual BAs such as DCA and *ω*MCA, while being negatively correlated with 3-oxoLCA and isoalloLCA (*P*<0.05, Fig. 5d).

## Discussion

Late gestation represents a critical stage in the reproductive cycle of sows, characterized by heightened metabolic demands [8] and increased susceptibility to oxidative stress and immune dysregulation [10,11]. Genetic background, as a key determinant of physiological potential, may profoundly influence host–microbiota interactions and metabolic phenotypes, yet the underlying mechanisms remain poorly understood. In this study, a comprehensive comparison of serum biomarkers, gut microbiota composition and BA profiles between LW sows and crossbred LW×LR sows was conducted. The findings provide novel insights into associations between genetic variation, gestational physiology and the ‘microbiota–metabolite axis’. This study demonstrated that, during late gestation, LW×LR crossbred sows showed increased serum activities of SOD, accompanied by decreased MDA levels. As MDA is a key marker of lipid peroxidation, its reduction directly reflects the alleviation of membrane lipid oxidative damage. These changes suggest that LW×LR crossbred sows possess a more robust antioxidant defence system capable of more effectively neutralizing reactive oxygen species and maintaining redox balance under increasing metabolic stress. In addition, the upregulation of IL-10, an anti-inflammatory cytokine, coupled with downregulation of the pro-inflammatory cytokine IL-6, suggests a shift toward an anti-inflammatory immune phenotype in LW×LR crossbred sows, consistent with previously reported hybrid vigour in immune resilience [37].

In this study, LW×LR crossbred sows demonstrated superior antioxidant capacity and a more favourable anti-inflammatory status during late gestation, which may reflect enhanced adaptive regulatory responses to increased metabolic demands. However, such physiological differences are unlikely to be solely attributed to the host genetic background. There is growing evidence that the gut microbiota, as a key regulator of host metabolism and immune function, may play an important intermediary role in shaping these outcomes [38,39]. Our gut microbiota profiling in late-gestation sows revealed significant structural differences between LW and LW×LR crossbred sows, suggesting a potential link between microbial composition and host phenotypic variation in oxidative and inflammatory responses. Crossbred LW×LR sows exhibited significantly higher *α*-diversity indices (Shannon) and a significantly lower Simpson index compared to LW sows. *β*-diversity analysis revealed distinct microbial community structures between the two groups, and LW×LR sows harboured a greater number of unique ASVs, indicating higher richness, diversity and specificity, which underscores the key role of genetic background in shaping gut microbiota composition. These findings align with previous reports. For example, studies have shown that the gut microbiota composition between Iberian and Duroc pigs differs more significantly than the microbiota variation caused by dietary changes within the same breed [40]. Similarly, the *β*-diversity patterns of gut microbiota in wild boar-Duroc hybrids were consistent with their genetic divergence [41]. In addition, hybrid lambs exhibited altered rumen microbial communities, including an enrichment of *Prevotella* and *Fibrobacter* genera, which was associated with enhanced carbohydrate-degrading capacity [42]. In addition, microbial stability was higher in LW×LR sows, as indicated by a greater microbial stability index [43]. Collectively, these findings underscore the critical role of genetic background in shaping microbial structure and function [40] and further imply a close association between microbial composition and host metabolic phenotype.

At the phylum level, LW×LR crossbred sows exhibited a higher relative abundance of Bacteroidota and a lower abundance of Firmicutes, resulting in a reduced *Firmicutes*/*Bacteroidota* (F/B) ratio. This shift has dual physiological implications. On one hand, increased *Bacteroidota* enhances the intestinal capacity to degrade complex carbohydrates, thereby providing metabolic support for the elevated energy demands during late gestation [44]. On the other hand, a lower F/B ratio is associated with reduced stress levels [45], which is consistent with the decreased MDA levels and downregulated inflammatory responses observed in LW×LR crossbred sows. These phylum-level features also lay the foundation for the enrichment of beneficial taxa at the genus level. Specifically, LW×LR crossbred sows showed notable enrichment of *Treponema* and *Prevotella*, all of which are functionally relevant. *Treponema* contributes to energy metabolism via fibre fermentation [46,47]. *Prevotella* helps maintain gut pH by producing short-chain fatty acids like succinate, which may suppress pro-inflammatory bacterial colonization and indirectly support anti-inflammatory effects [48]. The observed inverse correlations between IL-6 levels and the abundances of *Treponema* and *Prevotella* further suggest cooperative roles in modulating inflammation. Conversely, the enrichment of *Terrisporobacter* and *Clostridium sensu* stricto 1 in LW sows warrants closer attention. *Clostridium sensu* stricto 1 has been identified as a potential pathogenic genus associated with intestinal disorders [49,50] and has been reported to negatively correlate with piglet litter weight and average daily gain [51]. Additionally, elevated levels of *Terrisporobacter* have been shown to induce increased oxidative stress [51,52]. The enrichment of these taxa is consistent with the higher levels of inflammation observed in LW sows, suggesting a potential contributory role in promoting oxidative and inflammatory responses.

**Fig. 6.**
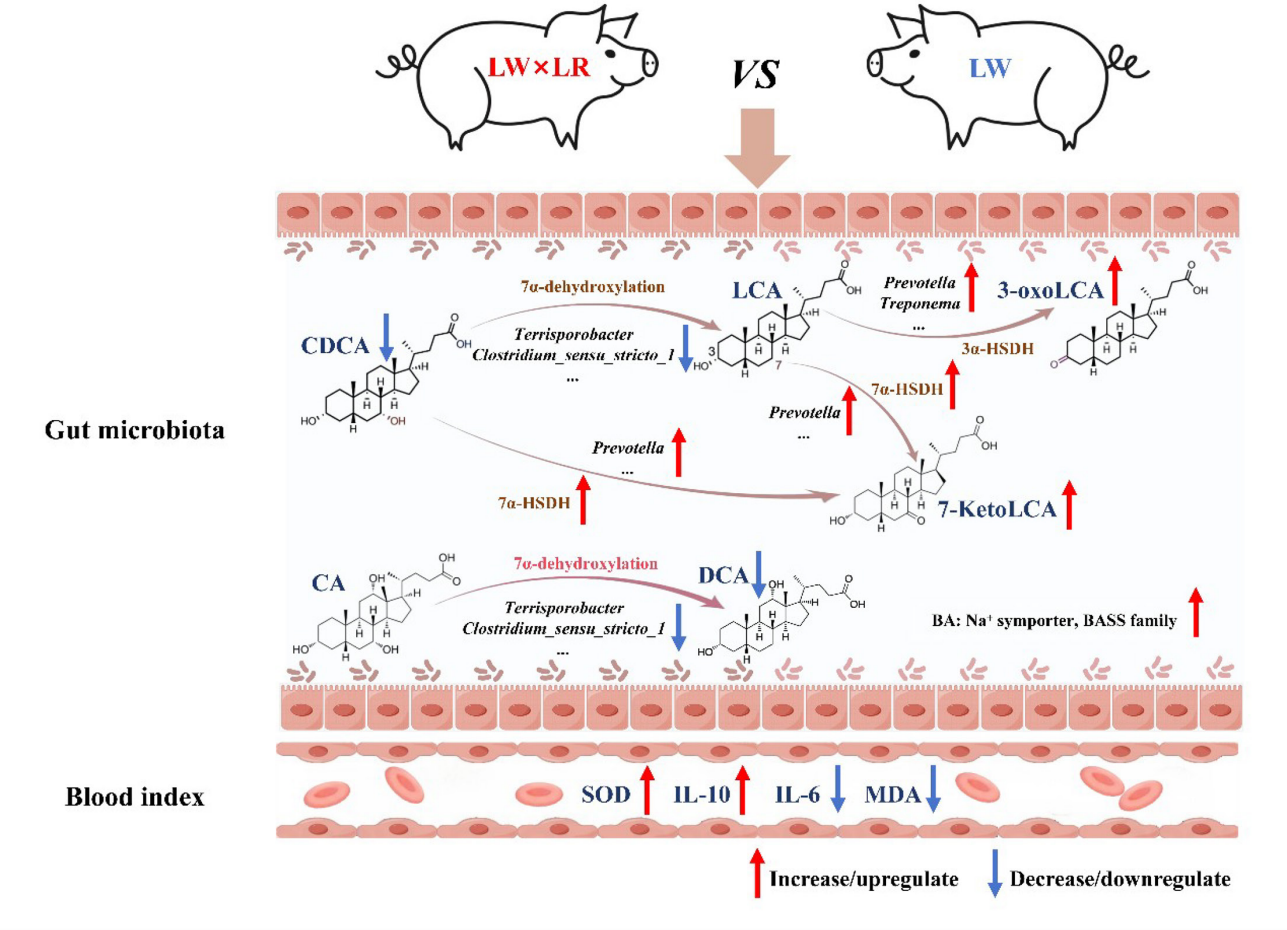
Schematic model illustrating the potential mechanisms underlying the differences between LW×LR crossbred sows and LW sows during late pregnancy with respect to gut microbiota, BA metabolism and host antioxidant and immune functions. In LW×LR crossbred sows, enrichment of BA-modifying bacteria (e.g. *Prevotella*), together with increased 7*α*-HSDH activity, promotes the conversion of LCA into 3-oxoLCA and 7-KetoLCA, while the upregulation of the BA: Na^+^ symporter (BASS family) enhances intestinal BA reabsorption and recycling. These changes are linked to enhanced antioxidant capacity (e.g. increased SOD activity) and improved anti-inflammatory response (e.g. increased IL-10 level), as well as decreased oxidative damage (e.g. lower MDA level) and reduced pro-inflammatory markers (e.g. reduced IL-6 level) in LW×LR crossbred sows. These findings highlight the pivotal role of genetic diversity in sustaining physiological stability and ensuring maternal health throughout pregnancy.

Functional prediction analysis revealed a significant upregulation of the BA: Na^+^ symporter (BASS family) in LW×LR crossbred sows, indicating enhanced intestinal BA reabsorption and recycling [53]. Concurrently, the genus *Prevotella* possesses potent BA-modifying capabilities. Members of this genus express 3α-HSDH, which converts LCA to 3-oxoLCA [54]. In addition, *Prevotella* can produce 7α-HSDH, an enzyme capable of directly or indirectly transforming CDCA into 7-ketoLCA [55]. These microbial transformations may enhance FXR activation and, thereby, support metabolic and immune homeostasis [56]. Together, increased host-mediated BA reabsorption and *Prevotella*-driven BA modification likely improve enterohepatic BA circulation efficiency and reduce faecal BA loss, consistent with the lower faecal BA levels observed in LW×LR crossbred sows. Enrichment of *Treponema* further reinforces the BA metabolic advantage, as it is positively associated with modified BAs (3-oxoLCA and 7-ketoLCA) and serum SOD activity and negatively associated with MDA levels. Together with *Prevotella*, *Treponema* may contribute to a transformation and reabsorption synergy, whereby the BA-modifying capacity of *Prevotella* [55], potential indirect effects of *Treponema* on BA modification [57] and the upregulation of BASS family proteins and 7*α*-HSDH collectively enhance the conversion of primary BAs into modified forms and their subsequent reabsorption, establishing a refined BA regulatory network that supports metabolic stability (Fig. 6). Although principal component analysis did not reveal a clear group separation in faecal BA composition, LW×LR sows exhibited a higher number of unique BA species (16 vs. 9), indicating more diverse BA metabolic networks. Among the BAs uniquely present in LW×LR crossbred sows, 3-oxoLCA was the most abundant. The 3-oxoLCA has been shown to exert key immunomodulatory effects by directly binding to the nuclear receptor RORγt, antagonizing its activity and suppressing the differentiation of pro-inflammatory Th17 cells, thereby contributing to intestinal immune homeostasis [58]. This immunomodulatory role of 3-oxoLCA, together with the antioxidant benefits associated with *Treponema* and *Prevotella*, helps explain the superior anti-inflammatory and redox balance phenotypes of LW×LR sows, which are consistent with their higher serum IL-10 levels and lower IL-6 levels observed in this study. In contrast, LW sows exhibit distinct challenges in BA metabolism. Their elevated faecal levels of both conjugated and unconjugated BAs may result not only from the high abundance of *Terrisporobacter* [59] and *Clostridium sensu* stricto 1 [60], but also from the inhibitory effects of these taxa on primary BA transformation. Correlation analysis confirmed that *Terrisporobacter* and *Clostridium sensu* stricto 1 are positively associated with primary BAs (e.g. CDCA and DCA), suggesting that they may hinder the conversion of primary BAs to modified forms, ultimately leading to primary BA accumulation and reduced production of beneficial modified BAs such as 3-oxoLCA. This metabolic impairment is further exacerbated by the relatively low expression of BASS family BA transporters and metabolic enzyme abundance in LW sows, which limits intestinal BA reabsorption efficiency and increases faecal excretion of conjugated BAs. The combination of accumulated unmodified BAs (e.g. DCA) and impaired reabsorption creates a pro-inflammatory environment [61,63]. DCA, a known pro-inflammatory BA [30,64], may stimulate the intestinal mucosa, while IL-6 levels, a key pro-inflammatory cytokine, are significantly positively correlated with *Terrisporobacter* and *Clostridium sensu* stricto 1 abundance and negatively correlated with functional genera such as *Prevotella*. Collectively, these findings indicate that BA metabolic disturbances in LW sows, driven by both microbiota composition and transporter activity, may contribute to their heightened inflammatory status. Such modulation may ultimately support gestational physiological homeostasis during late gestation in sows, offering new insights into the microbial basis of crossbred sow physiological advantages.

While this study identifies meaningful associations, such as the correlation of *Treponema* with modified BAs and serum SOD activity, and offers preliminary insights into gut microbiota–BA–antioxidant crosstalk in sows, key limitations should be noted to avoid overextrapolation. First, all sows were sourced from a single commercial farm. Given that gut microbiota is shaped by factors like cohabitation and management [65], and this pattern is likely relevant to livestock, the observed taxonomic patterns (e.g. *Treponema* enrichment in crossbred sows) may not apply to other farms with different conditions. Second, conclusions about taxa regulating BA metabolism or antioxidant activity remain speculative: no intervention experiments were conducted to confirm causality, so correlational findings are associative rather than definitive. Future work will address these gaps by expanding sampling to multiple farms (to test generalizability) and designing intervention trials (key microbial colonization) to validate causal links. This will strengthen the translational value of our findings for sow health.

## Conclusion

This study highlights the impact of genetic background on pregnancy adaptation through the microbiota–BA–host axis in late-gestation sows, under the experimental conditions of standardized intensive production (individual gestation stalls) and a gestation-phase complete diet formulated to meet NRC (2012) nutrient requirements. Compared to LW purebred sows, LW×LR crossbred sows demonstrated enhanced antioxidant and anti-inflammatory capacities, enrichment of beneficial gut microbiota taxa (e.g. *Prevotella*), elevated levels of regulatory BAs (e.g. 3-oxoLCA), upregulated BA transporters and metabolic enzyme abundance (7α-HSDH), collectively supporting metabolic and immune stability. In contrast, LW sows exhibited enrichment of pro-inflammatory taxa and BA accumulation, which may compromise physiological homeostasis. These findings reveal gut microbiota–BA–host associations underlying the physiological advantages of crossbred sows within the studied production and dietary context, emphasizing the critical role of genetic diversity in supporting gestational physiological stability and health.

## Supplementary material

10.1099/mgen.0.001579 Uncited Supplementary Material 1.

## Abbreviations

apoCA: apocholic acid
ASVs: amplicon sequence variants
AVD: average variation degree
BA: bile acid
C4: 7*α*-hydroxy-cholestene-3-one
CAT: catalase
CDCA: chenodeoxycholic acid
DCA: deoxycholic acid
DCA-3S: deoxycholic acid-3 sulphate
dehydroLCA: dehydrolithocholic acid
DHCA: dehydrocholic acid
6,7-DiketoLCA: 6,7-diketolithocholic acid
ESI: electrospray ionization
F/B: *Firmicutes*/*Bacteroidota*
FXR: farnesoid X receptor
GCA: glycocholic acid
GDHCA: glycodehydrocholic acid
GHDCA: glycohyodeoxycholic acid
GLCA: glycolithocholic acid
GUDCA: glycoursodeoxycholic acid
HCA: hyocholic acid
HDCA: *α*-hyodeoxycholic acid
isoalloLCA: isoallolithocholic acid
KEGG: Kyoto Encyclopedia of Genes and Genomes
6-KetoLCA: 6-ketolithocholic acid
7-KetoLCA: 7-ketolithocholic acid
12-KetoLCA: 12-ketolithocholic acid
LCA: lithocholic acid
LCA-3S: lithocholic acid-3 sulphate
LW: large white
LW×LR: large white×landrace
MCA: muricholic acid
MDA: malondialdehyde
muroCA: murocholic acid
NCBI: National Center for Biotechnology Information
NCM: neutral community model
NorCA: norcholic acid
NorDCA: nordeoxycholic acid
NRC: National Research Council
3-oxoLCA: 3-oxolithocholic acid
PCA: principal component analysis
PCoA: principal coordinate analysis
SOD: superoxide dismutase
T-AOC: total antioxidant capacity
TDHCA: taurodehydrocholic acid
THCA: taurohyocholic acid
THDCA: taurohyodeoxycholic acid
TLCA: taurolithocholic acid
TNF-*α*: tumour necrosis factor-*α*
T*α*MCA: tauro-*α*-muricholic acid
T*β*MCA: tauro-*β*-muricholic acid
UCA: ursocholic acid
UDCA-7S: ursodeoxycholic acid-7 sulphate
3*α*-HSDH: 3-*α*-hydroxysteroid dehydrogenase
7*α*-HSDH: 7-*α*-hydroxysteroid dehydrogenase
*α*MCA: *α*-muricholic acid
*β*DCA: *β*-deoxycholic acid
*β*HDCA: *β*-hyodeoxycholic acid
*β*MCA: *β*-muricholic acid
*β*UDCA: 3*β*-ursodeoxycholic acid
*ω*MCA: *ω*-muricholic acid
