# Supplementary material for: Gut microbiota and bile acid profiles in purebred vs. crossbred sows: links to oxidative stress and inflammation in late gestation
Source: Microb Genom. 2025 Dec 3;11(12):001579. doi: 10.1099/mgen.0.001579 (PMC13293294; doi:10.1099/mgen.0.001579)
Supplement: Uncited Supplementary Material 1. [file mgen-11-01579-s001.pdf]

Supplementary Tables and Figures

Table S1. NCBI biological sample serial number

Data Repository	BioProject Accession Number	Groups	Biosample Accession Numbers
NCBI SRA	PRJNA1295093	LW	SAMN50148236, SAMN50148237, SAMN50148238, SAMN50148239, SAMN50148240, SAMN50148241, SAMN50148242, SAMN50148243, SAMN50148244, SAMN50148245, SAMN50148246, SAMN50148247, SAMN50148248, SAMN50148249, SAMN50148250, SAMN50148251, SAMN50148252, SAMN50148253, SAMN50148254, SAMN50148255.
NCBI SRA	PRJNA1295093	LW_LR	SAMN50148256, SAMN50148257, SAMN50148258, SAMN50148259, SAMN50148260, SAMN50148261, SAMN50148262, SAMN50148263, SAMN50148264, SAMN50148265, SAMN50148266, SAMN50148267, SAMN50148268, SAMN50148269, SAMN50148270, SAMN50148271, SAMN50148272, SAMN50148273, SAMN50148274, SAMN50148275, SAMN50148276, SAMN50148277, SAMN50148278, SAMN50148279.

Table S2. Ingredient compositions and nutrient levels of gestation diets used (%, as-fed basis).

**Items**	**Gestation**
Ingredients	
Corn	67.69
Soybean meal, 46% CP	14.00
Wheat bran	14.00
Soybean oil	1.60
Sodium chloride	0.40
Limestone	1.13
Calcium dihydrogen phosphate	0.57
Phytase	0.04
Choline chloride, 60%	0.07
Vitamin and mineral premix[1]	0.50
Total	100.00
Analyzed nutritional value	
Crude protein, %	14.30
Calcium, %	0.62
Phosphorus, %	0.50
Ether extract, %	2.97
Crude ash, %	4.80
Calculated nutritional value[2]	
Metabolizable energy, kcal/kg	3108
Lysine, %	0.63
Methionine + cysteine, %	0.48
Threonine, %	0.51
Tryptophan, %	0.16

[1] The premix supplied the following vitamins and trace minerals per kilogram of diet: Cu, 15 mg; I, 0.3 mg; Mn, 50 mg; Se, 0.3 mg; Fe, 80 mg; Zn, 100 mg; vitamin A, 25,000 IU; vitamin D_3, 5,000 IU;

vitamin E, 50 IU; vitamin K, 2.5 mg; biotin, 0.2 mg; vitamin B_1, 1.0 mg; vitamin B_2, 8.0 mg; vitamin B_6, 3.0 mg; vitamin B_{12}, 0.020 mg; niacin, 15.0 mg; pantothenic acid, 12.5 mg; folacin, 1.50 mg.

[2] The calculated nutritional values were based on the Nutrient Requirements of Swine (NRC, 2012) guidelines.

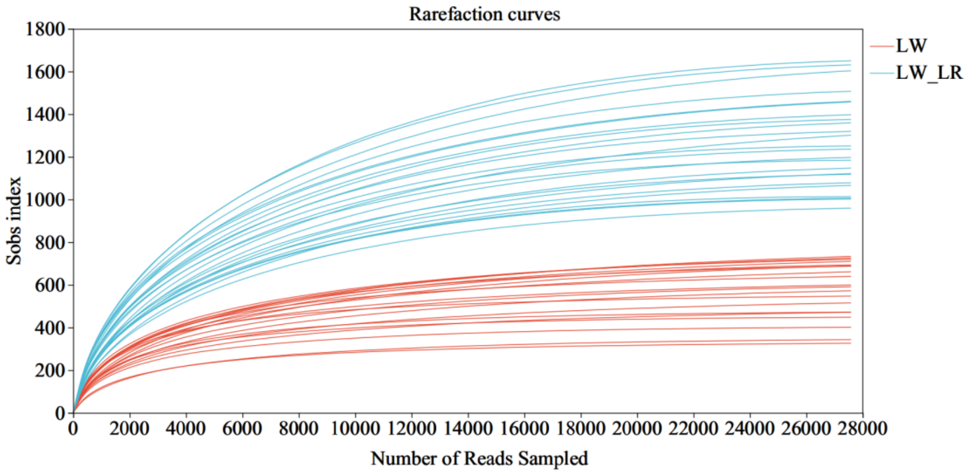

Fig. S1. Rarefaction curves of fecal microbiota from LW and crossbred LW×LR sows during late gestation. LW, Large White sows; LW_LR, Large White × Landrace crossbred sows.

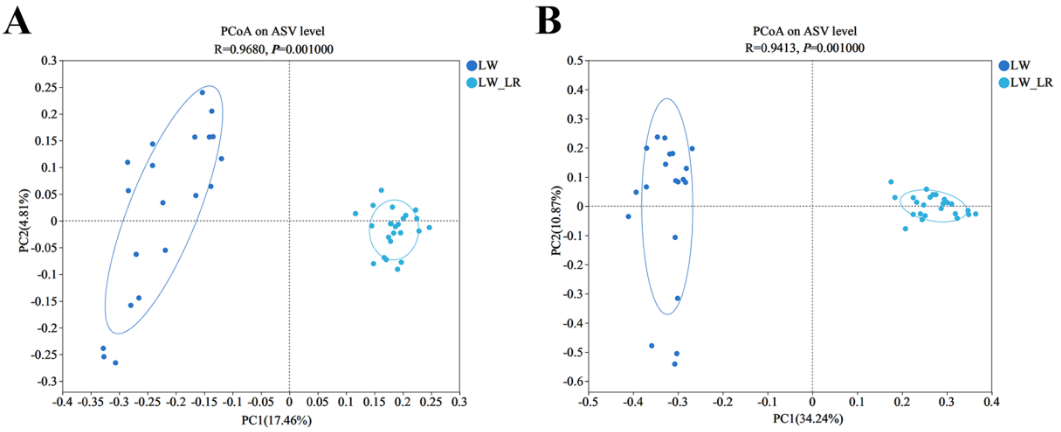

Fig. S2. Principal coordinate analysis (PCoA) was performed based on unweighted unifrac (A) and Bray-curtis distance (B) at the ASV level to assess β-diversity of fecal microbiota from LW and crossbred LW×LR sows during late gestation. LW, Large White sows; LW_LR, Large White × Landrace crossbred sows.

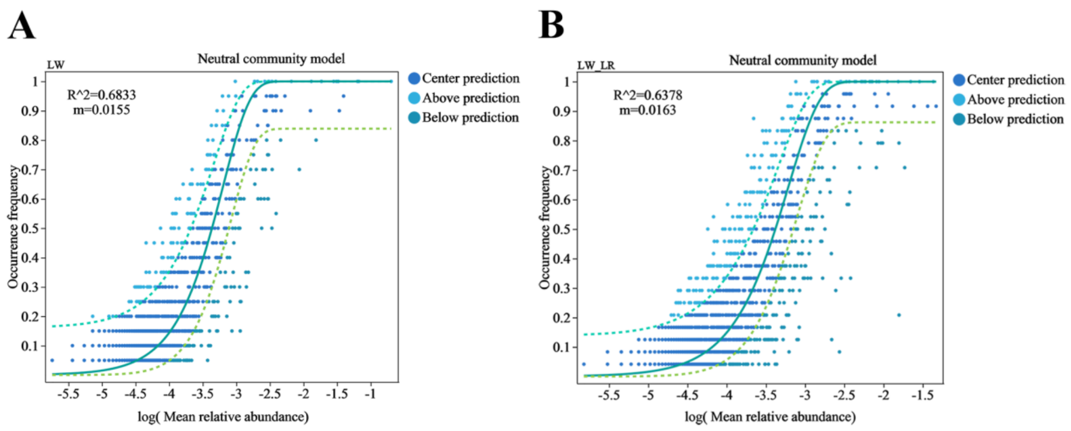

Fig. S3. The microbial structure analysis based on the neutral community model (NCM). (A) Analysis results for fecal microbiota from LW sows; (B) Analysis results for fecal microbiota from crossbred LW×LR sows; LW, Large White sows; LW_LR, Large White × Landrace crossbred sows.

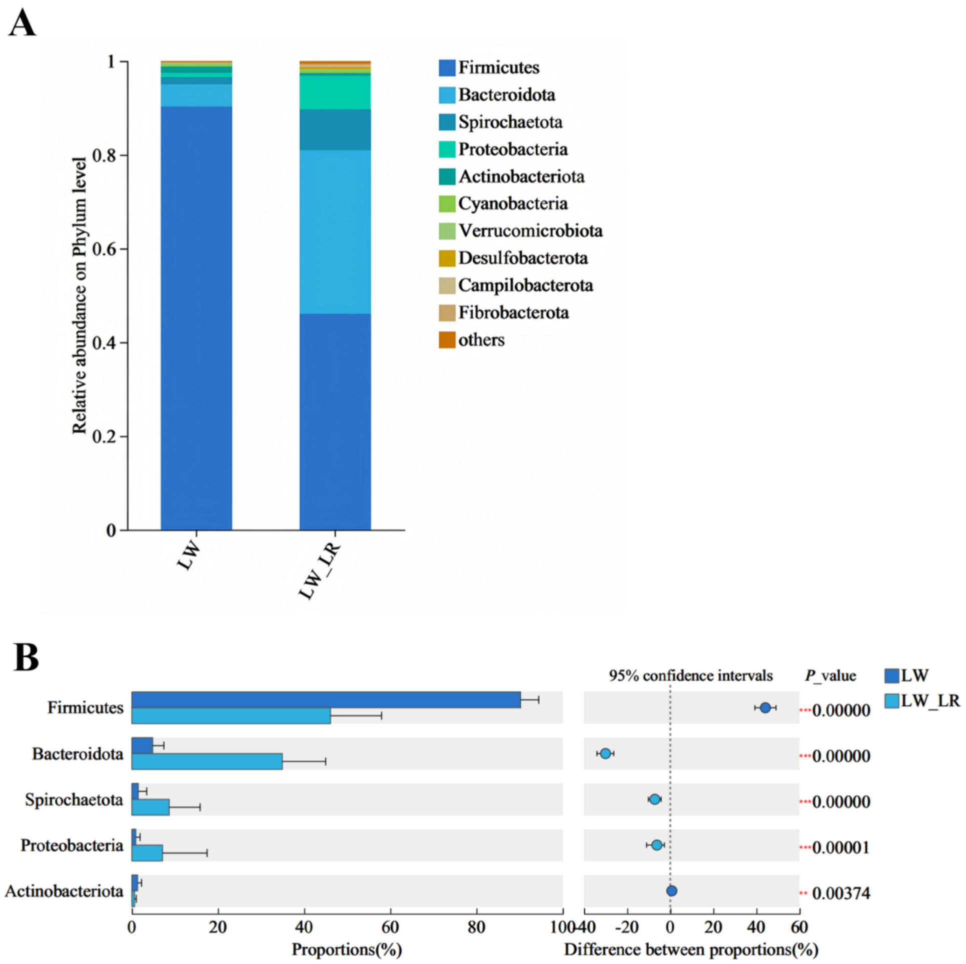

Fig. S4. Phylum-level composition (A) and differential analysis (B) of fecal microbiota in LW and crossbred LW×LR sows during late gestation. LW, Large White sows; LW_LR, Large White × Landrace crossbred sows.

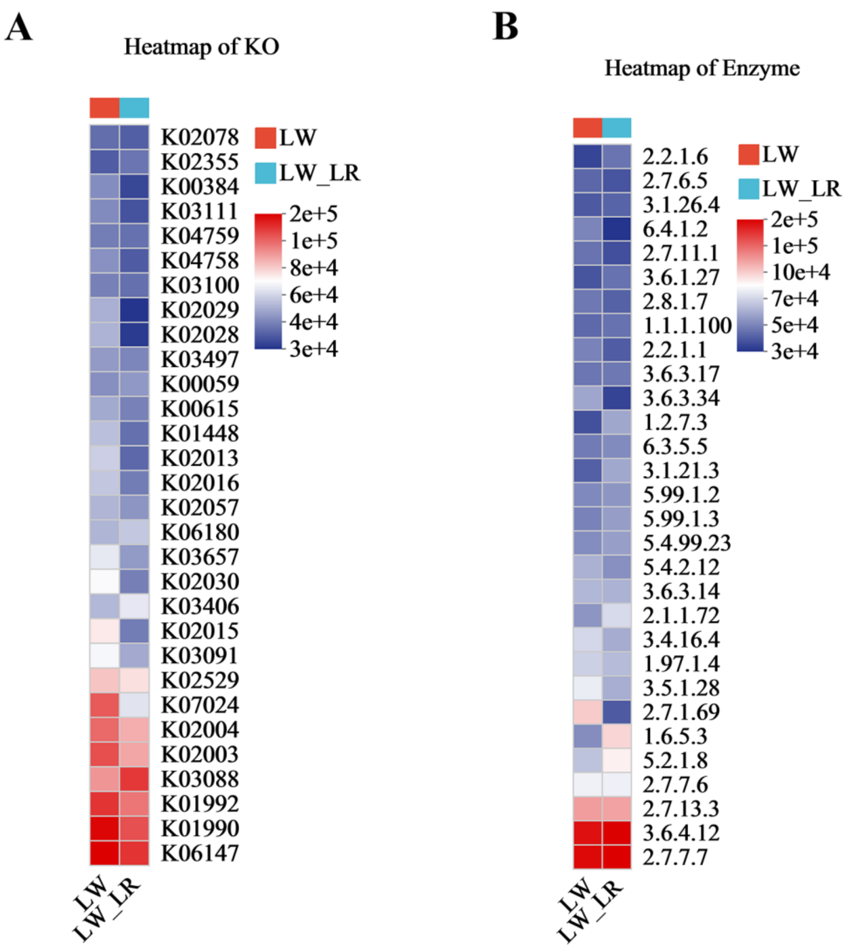

Fig. S5. PICRUSt2-based prediction of microbial pathway abundances (A) and enzyme abundance (B) in LW and crossbred LW×LR sows during late gestation. LW, Large White sows; LW_LR, Large White × Landrace crossbred sows.
